# Perceived impact of formulating, implementing and enacting national mental health policies recommendations in practice: An exploratory qualitative study within child and adolescent mental health services in Scotland

**DOI:** 10.1177/13558196211072472

**Published:** 2022-02-28

**Authors:** Madalina Toma, Julie Anderson, Sarah Forster, Paula Shiels, Shirley Windsor, Nicola M Gray

**Affiliations:** 1 NIHR Applied Research Collaboration (ARC) Kent Surrey Sussex, Personal Social Service Research Unit (PSSRU), School of Social Policy, Sociology and Social Research, University of Kent, Canterbury, UK; 2 Performance, Planning and Quality Manager, 130121 NHS National Services Scotland, Edinburgh, UK; 3 Head of Service Design and Transformational Change, 102585 Aberlour Childcare Trust, Stirling, UK; 4 Senior Nurse Mental Health, 1442 NHS Ayrshire & Arran, Crosshouse, Kilmarnock, UK; 5 Organisational Lead Public Mental Health (Implementation), 578987 Public Health Scotland, Edinburgh, UK; 6 Scottish Improvement Science Collaborating Centre (SISCC), School Health Sciences, 3042 University of Dundee, Dundee, UK

**Keywords:** children and young people, mental health, national policies

## Abstract

**Objective:**

To understand the process of formulating, implementing and enacting national recommendations into practice, by exploring the interactions between government policymakers and national and local organisations supporting and delivering policy implementation within a Child and Adolescent Mental Health Service (CAMHS) context in Scotland.

**Methods:**

Data collection involved 16 semi-structured individual and four focus group interviews with a purposeful sample of policymakers, national health and social care stakeholders and local outpatient and inpatient CAMHS teams representing three NHS health boards in Scotland.

**Results:**

Study participants highlighted the challenges of navigating through evolving and often conflicting policy agendas, seen to not acknowledging the current evidence base or experiential learning from services and prior evaluations. Accounts of transformation fatigue often emerged from increased expectations for staff to adopt new approaches to accommodate constantly changing recommendations. Participants also reported a lack of integration and implementation support from national health and social care organisations, leading to duplication of effort and gaps in provision or waste. Policy recommendations were perceived as sometimes vague, lacking clarity about how to deliver service transformation using a whole-system approach. The collective narratives reflected increased tension between the need for local autonomy to innovate and the limitations created vertically by the relative inflexibility of policy recommendations, and horizontally by the proliferation of national organisations delivering the same transformation aims using different approaches in a resource-constrained environment.

**Conclusion:**

The findings contribute to the wider literature by offering an exploration of importance of evaluation and evidence uptake in policy formulation; the roles and remits in supporting the implementation of policy recommendations; and how the dynamics of central control and local autonomy might impact on the local enactment of policy recommendations.

## Introduction

Mental health is one of the leading global public health challenges as measured by prevalence, burden of disease and disability.^1^ Children and young people’s (CYP) mental health has become an international priority and there is growing interest in how to alleviate the additional risks of developing comorbidities and subsequent life-long consequences of early onsets.^2-4^ As the prevalence of mental health issues increases, so does the number of CYP trying to access specialised services, putting pressure on already strained mental health services worldwide.^5^ In Scotland, United Kingdom (UK), the National Health Service (NHS) provides Child and Adolescent Mental Health Services (CAMHS)^6^ to diagnose and treat CYP mental health problems that require specialist level interventions, care and support. Around 10% of CYP in Scotland have a diagnosable mental health disorder, with 50% of mental health problems established by the time young people turn 14 years old.^7^ A 22% rise in the number of new referrals between 2013/14 and 2017/18^8^ has placed the health and social care system under increasing pressure, leading to long waiting times for outpatient services, an increase in demand for inpatient care, and complicated care pathways with significant variation of service delivery models across Scotland.^9,10^

In response, the Scottish government published, in 2017, a 10-year Mental Health Strategy.^11^ The strategy seeks to improve the mental health of Scotland’s population, including CYP, taking a multi-disciplinary system-wide approach by promoting the integration of public and third sector CYP mental health services with a clearly defined focus on prevention and early intervention. System-wide challenges as identified by the strategy and subsequent reviews include a ‘complex and fragmented’ system of mental health services experienced by CYP and their families, with ‘patchy and inconsistent’ referral pathways, leading to one in five CAMHS referrals being rejected as inappropriate.^12^ Other work confirmed substantial variability in access to CAMHS across Scotland^8^ and gaps in frontline provision, leading to CYP often facing barriers to accessing appropriate help at the right time.^13^ A CAMHS taskforce has since been set up, which, among other things, is developing recommendations for a blueprint for how children and young people’s services should support the transformational change to improve the organisation of mental services in Scotland, and in partnership, develop a programme of sustainable whole system reform locally.^14^

However, despite widespread support for implementing the Scottish Government’s Mental Health Strategy and improving CAMHS more widely, translating needed transformational change into practice is fraud with challenges.^15^ The determinants of ‘what works’ in achieving change are complex and the extent to which policy is translated into sustained local improvements is not yet fully explored in this setting, representing in many ways a ‘missing link’ in the study of similar policies.^16^ This study seeks to help fill this gap by exploring the perceived impact of formulating, implementing and enacting recent national mental health policy recommendations into local practice, identifying the key issues needed to support the change needed to achieve meaningful improvements within CAMHS in Scotland.

## Methods

Data were collected during January–September 2019, using documentary analysis, individual interviews and focus group interviews. We first carried out a brief documentary analysis of key strategic and operational CAMHS-relevant policy recommendations in Scotland. This served to frame the subsequent qualitative data collection.

### Semi-structured interviews

Interview participants were identified through policy documentation, web searches and relevant networks of the project team. A participant recruitment mapping template was created which aimed to illustrate and clarify key individuals and organisations involved in either developing mental health policies or commissioned to support the delivery of policy recommendations into practice across Scotland. After populating the template with relevant stakeholders and their contact details, we invited 25 potential participants by email and, if unavailable, asked for suggestions for an appropriate replacement. The final sample included a combination of purposive and snowball sampling techniques.

Interviews were semi-structured in nature, and carried out by using an interview schedule informed by the literature in the field and, iteratively developed in two pilot interviews with academic experts in mental health policies (Online Supplement 1). All interviews were conducted in a non-directive manner by MT, who is an experienced qualitative health services researcher. Interviews were conducted face to face or by telephone and lasted between 40 and 75 min. Informed consent was obtained from all participants prior to the actual interviews.

### Focus group interviews

Focus group participants were purposefully selected from three NHS health boards in Scotland. and included teams who delivered inpatient and outpatient services. They were identified through internal networks and partners, and other study participants. Focus groups were facilitated by two experienced moderators (MT, JA) whose main roles were to encourage open and relevant discussions, probing areas for clarification. Discussions lasted approximately 90 min. The semi-structured interviews described above played an interim role in refining the questions and probes used during the meetings (Online Supplement 1).

### Analysis

All individual and focus group interviews were audio-recorded using an encrypted digital recorder, transcribed verbatim by an authorised professional company and analysed using NVivo11. The lead author (MT) coded all interview data, with five randomly selected transcripts independently reviewed by two other experienced researchers to refine the codebook and incorporate new codes. Differences were identified and resolved by consensus discussion within the research team. Interview analysis followed the principles of the framework approach,^17^ which facilitated the development of an iterative and stepwise coding matrix, which was subsequently elaborated on during the focus group interviews. MT also coded focus group data and the research team met regularly to reach consensus on the final framework structure, discuss additional categories, and resolve disagreements.

We used meta-matrix triangulation technique^18^ to corroborate themes identified from the focus groups with interview data. By sequencing data collection and analysis in this way, it was possible to uncover patterns, convergence or contradictory experiences which further enriched the conceptualisation of how mental health policy recommendations are formulated and implemented in practice.

## Results

### Participant characteristics

We conducted 16 individual semi-structured interviews and four focus group interviews with a total of 22 participants. Interview participants were national policymakers from across the Scottish Government Mental Health Directorate involved in monitoring performance and setting the general direction of quality improvement within mental health (*n* = 7) and stakeholders representing statutory organisations and the voluntary sector with a national and strategic position in developing or advising on mental health policy priorities and oversight of the decision-making processes relating to the local implementation of national policies (*n* = 9). Identified narratives were similar across interview participants, which suggested that additional interviews would bring limited new insights.

Focus group interviews were conducted with three local outpatient and one inpatient CAMHS teams; participants’ roles and professions reflected the nature of the service’s cross-sectoral approach to providing services and meeting local needs. The overall sample included five CAMH service managers, five improvement advisors, three CAMHS nurses, two consultant psychiatrists, two clinical psychologists, two occupational therapists, one general practitioner, one headteacher and one third sector representative. All members of the multi-disciplinary team were involved in delivering improvements locally but also retained a significant role within their routine clinical practice.

In what follows we report findings from our analysis under three headings: evaluation and evidence uptake in policy formulation; roles and remits in supporting the implementation of policy recommendations; and dynamics of central control and local autonomy in the enactment of recommendations.

### Evaluation and evidence uptake in policy formulation

Several participants described the lack of fidelity with previous directives and questioned whether the recently emergent policies took cognisance of, and were formulated on, relevant and credible evidence and evaluations of previous policy and practice initiatives. For instance, concerns were raised about the historical reluctance to test and evaluate policies and the setting of new objectives without addressing the lessons learned from previous successes and failures.

We talk about now as being the golden age of mental health policy, but I have been around for long enough to remember towards the mentally flourishing Scotland, when See Me [2001], Choose Life [2002] and the Scottish Recovery Network [2004] started. There were really good bits of inclusive policies…but it just all vanished, and I do not know where are the lessons that we learned and the key messages taken forward. Also, it is baffling that we find ourselves now, five/six years on from the last mental health strategy, with a brand new one without any thought about what had happened previously. Almost like we are counting on the fact people have short memories and we can just quietly park the things that we did not achieve and come up with new recommendations that we assume to be beneficial with no evidence. *(Improvement advisor)*

Furthermore, while the overall policy intentions were welcomed, participants questioned whether there was scientific evidence supporting the most recent Mental Health Strategy and follow-up recommendations.

I do not know where the 18 weeks referral to treatment numbers came from, but what’s interesting is that it is 18 for mental health but for other services it is 12 also if you were psychotic 18 weeks is nonsense. If you are in hospital having taken an overdose as a young person you should be seen within 24 hours…if you were asking a parent, they’d tell you four months to wait is completely outrageous. Anyone that has lived with a mental health condition will tell you 18 weeks is wild and feels like that’s a lifetime for a person in need. *(National stakeholder)*

In contrast, although policymakers interviewed in this study agreed that evidence clearly contributes to thoughtful policymaking, they believed that evidence should inform but not drive political decisions.

I am not familiar about why they chose a particular timeline and target you know, why they chose the 90% [18 Weeks Referral to Treatment standard should be delivered for at least 90% of patients], all these things, I do not have the history of the evidence behind it. What I can definitely tell you is that evidence surely must inform this process, but, equally, it cannot be decisive in driving policy decisions. When we make decisions, we are driven by a desire to achieve a set of goals. The role of evidence is to support how our choices are likely to affect the realization of our goals so that, if the evidence is good and we interpret it well, the results of our decisions align better with what we value. *(Policymaker)*

The pace, direction, and urgency of the new recommendations set out in the Mental Health Strategy, coupled with a lack of robust utilisation of evidence and evaluation was a challenge many frontline staff reflected on. Some participants described transformation fatigue, resulting from constant expectation of improvement of services to meet increasing levels of demand, with very little learning from the past.

You have got a policy initiative coming from government around children’s mental health, wanting to reduce waits, wanting case access to services and we want you to do this and that. But you have just delivered on one target and now a completely new one is enforced. Halfway through your transformation of services you have to stop. That’s where mixed messages comes in, you can see a lot of tiredness around, ‘Okay, how are we gonna do this? ‘cause by the time that we come to terms with what needs to be done, there’s a new government with a new agenda, with new priorities, and then we start again with no time to think about what we did in the past. It just feels like you just cannot get off that transformation treadmill and cannot keep on top of things. Let us not forget that the workforce in specialist CAMHS is whole time equivalents of about 11 hundred people who are constantly blamed not changing and adapting quick enough. *(Occupational therapist)*

### Roles and remits in supporting the implementation of policy recommendations

Many participants described the move towards national level implementation support, with the involvement of national organisations across the public sector spectrum. These national organisations were often commissioned by the government to directly deliver policy aims and objectives, with some having a dual role to also support the national implementation of these policies. The focus of the support provided by these organisations varied substantially from the formulation of recommendations, to producing national guidelines and good practice resources. However, the unanimous view was that there was little integration and cohesion between the broad spectrum of national organisations and their activities, leading to an abundance and overlap of implementation support and organisations delivering the same transformations aims but using different approaches.

You could easily be disrupted by all the stories going on. You have got the recommendations from the mental health strategy, the recommendations coming from the [anonymised organisation], the audit of rejected referrals and so on. It is a bit of a mish mash at the minute, a massive overlap which is still to be addressed if we want to avoid fragmentation from the policy end of things. We are doing so much around mental health in lots of different ways and across lots of recommendations that somehow, we are not factoring [in] what that looks like in its entirety. It is difficult to keep track some of these things making sure you are always linked up and up to date with all these recommendations. *(General practitioner)*

### Dynamics of central control and local autonomy in the enactment of recommendations

Policymakers noted that policy recommendations were developed to set a strategic direction of travel and achieve an overall objective. They described how they sometimes deliberately offered little guidance around operational implementation in order to enable flexibility from individual services to implement the objectives relevant for their own local populations.

Policy tends to be loose enough to be able to change and adapt, because of the world in which it works is messy. This strategy [Mental Health Strategy 2017–2027] is what you might call expressions starter for ten, something that will be very important to consider when you are further taking forward your own locality-based work. We think of the strategy as the starting point that sets the framework, it says broadly what are the areas where activity is needed, what we need to change but it is not a final statement of the all the things that have to happen. No matter what we do it is a national health service and the recommendations have to be somehow flexible so they can apply to individual lives both in the Borders or [in] the Highlands of Scotland. *(Policymaker)*

However, this approach to formulating recommendations was perceived very differently by local CAMHS teams. For some, the lack of specificity left too much room for interpretation locally, leading to confusion and ambiguity around the policy aims and objectives, particularly around what the specific policy recommendations mean in relation to service transformation and delivery.

A lot of the things that are in the current mental health policies, the forty, fifty, sixty odd recommendations, are so broad brushed, are too generic, too vague, too nicey-nicey, too wide reaching in terms of prevention, early intervention, and so on. So, it feels like more of the same, fairly common sense rather than giving an indication of how specific aims can lead to transformation in practice. The key problem, is that how are all these objectives all filtered down into a Service Manager and staff level of understanding? How is this going to help me with my job? You have produced guidance, or you come up with a national recommendation you need to actually provide some support on the ground about interpreting some of that. *(Service manager)*

Another example highlighting the challenges within the dynamic of central policies and local implementation is around the promotion of cross sector partnerships to achieve the desired transformation of mental health services. This acknowledges that children’s mental health and wellbeing is provided by health and non-health services and systems. However, despite a political rhetoric of empowering stakeholders, participants consistently reported that there was little guidance as to how different sectors can truly influence and transform services in a coordinated way. The recommendations around integrated working were considered exceedingly vague and CYP still received fragmented services because of lack of communication between parts of a system and between systems.

Joining mental health across health and social care is the priority from the government whole system approach, but there are too many partners at the table and the message sounds fragmented. I think there’s still some definite professional silos where people are focussed on their own pot of what’s going on and not actually making the connection between all these different aspects of mental health. The issue for me is that we need to find a way to captures all the key elements of specialist mental health along with the work that’s being done in education, third sector, work with families, primary care…something that pulls everything together to create a unified vision in Scotland. *(National stakeholder)*

In contrast to the view that many of the recommendations of the Mental Health Strategy were too vague, other narratives suggested that when recommendations were too specific, they did not chime well with the establishment of organisational and individual autonomy. The level of specificity often inhibited innovation as services approached improvement as something done to them, rather than by them.

I have lived through transformation two or three times, and if you are sitting waiting on direction from government it will never happen. Rather than the command and control from top down, we need to step up, put our head above the parapet and have some flexibility to determine what best [meets the] needs for our local community. There is probably an element of experimentation, but you do not just wait for things to be done for you, and to you, and expect the government to tell you how to run your service. Probably one of the best compliments I have ever had was from someone from the government who said it in a very doctorly kind of, ‘Who gave you permission to do that?’ And I said, ‘No-one.’ She said, ‘That’s what I wanted to hear.’ *(Third sector representative)*

Similar examples included the risk of tunnel vision as a consequence of setting specific waiting time targets for CYP who need specialist mental health services. There was a perception that these government set targets and recommendations shifted the focus and behaviours onto delivering the short-term goals, rather than addressing the fundamental issues within the services that need to transform in order to deliver personalised holistic care in the long-term.

You are having to model the service around the target, as opposed to needs of the children and you cannot see the wood for the trees. Simply, those targets drive the wrong behaviours, they are like a double-edge sword. Sometimes we become so fixated about fixing, to publicly been seen to have made a dent in the waiting list or something else that’s tangible or quantifiable, that we actually forget to look at the bigger picture. And the consequence of meeting the 18 weeks referral to treatment is that you are gonna have to tighten your referral criteria to meet a percentage. But that’s only one part of the puzzle…to meet that percentage, some kids are getting turned away from the service because of what the government asked us to do. *(Specialist nurse)*

## Discussion

This study illustrates that the process of mobilising policy into practice is not always straightforward. It highlights the importance of understanding how the formulation of policy and its implementation structures might impact on enactment and uptake in practice, and how to account for inherent trade-offs when implementing policy recommendations in a way that meets the increasing demands and local needs.

Policy recommendations are often released within a specific governance setting, are dependent on the context and the audience being addressed and are likely change over time. Study participants argued that evidence should shape the eventual goals of the policy agenda, yet there was a strong and consistent overarching theme running through the respondents’ narratives that there was insufficient use of up to date high-quality evidence, and a lack of rigorous testing and evaluation which limited what could be learnt from past successes and failures. This is consistent with literature on good governance of evaluation in policy development, including interactions between policymakers and researchers and timely availability of empirical evidence.^19,20^ However, qualitative or qualitative evidence cannot, by itself, determine policy and systems decisions, and therefore conceptualising how evidence could be used in a way that is politically appealing is complex. There might be a need to draw upon formative and summative evaluation to identify policy impacts and improve the overall understanding of what routine policy appraisals and high-quality evidence might entail. The idea that policy should be informed by evidence, but cannot be derived from it, raises important questions about how we should evaluate evidence use by decision makers. What constitutes a ‘good’ use of evidence in the context of mental health policy-making is also a controversial topic, with recommendations highlighting the appropriateness, transparency, accountability and contestability of data.^21^

Lack of clarity around the evidence base for some policy recommendations, in conjunction with the lack of appraisal of previous policies, initiatives and service experiences, was seen to put additional pressure on frontline staff, who struggled to navigate often conflicting policy agendas and adopt new mindsets and approaches. Across some interview participants this has led to a considerable level of the exhaustion and fatigue. This extent of this ‘fatigue’ is rarely evaluated systematically, and there are major technical challenges to doing so. There are - but substantive, ethical and moral reasons why policymakers should adopt a more balanced view of the positive and negative policy enactment unfolding spirals, which can produce either success or failure.^22^

In addition to the challenges of delivering local transformation, most participants in this study described a cluttered landscape of national health and social care organisations tasked with bridging the understanding between national and local narratives and delivering policy recommendations. These “intermediary organisations”^23^ work alongside and often at the direction of government and appear to play a critical role not only in implementing policy recommendations, but also in developing the necessary local capacity for system change. However, in the absence of a coherent well-articulated national vision, participants in this study reported a lack of integration and cross-fertilisation of information and knowledge sharing, as well as a duplication of efforts and emerging recommendations which created either gaps in provision, or waste. A major challenge for the future remains the vertical and horizontal coordination of the role of national organisations and alignment of improvement agendas and resources that move away from the traditional tiered approach to mental health and bring together public and third sector CYP services through a more extensive whole system approach to change.^24^ It will therefore be important to develop forums for collaborative policy commissioning and networks that allow an effective flow of information between all intermediary organisations early within the policymaking process. To this end, more work needs to be done to create a ‘national lever’ that clarifies complementary roles and lines of responsibility needed to provide the necessary political and social basis for building a coherent mental health agenda.^25^

The language of some policy recommendations was described as deliberately vague on substance, detail and action. Policy narratives for change are indeed often formulated at national level to ensure some degree of consistency in delivery while some space for manoeuvre is needed to shape and tailor recommendations to fit to local contexts and enact them within practices.^26^ This vagueness allows policy statements to act as expressions of intention rather than as deliberate courses of specific action. However, this was perceived in rather contradictory ways by study participants. Some commented on the lack of clear, specific, policies that identify clear pathways for service transformation. Participant narratives highlighted that little guidance was given on how health and social care, mental and physical health care or the acute and community care sectors can truly and equally contribute to establishing the direction of national transformation, in a coordinated way. At the same time there was a general agreement that if the recommendations are too directive, they might hinder innovation, leaving services unable to provide tailored solutions based on evolving needs, expectations and preferences of local populations.

The relative inflexibility of the policy process was seen to leave little room for stimulating innovation, and of particular concern was the increasingly bureaucratised and less personal nature of services, risking tunnel vision on delivery of specific goals. Examples include waiting time targets, with the focus on ensuring CYP are seen for their initial referral within a given time frame risking to remove resources from follow-up care and causing harm at different stages along the patient pathway. This is particularly important as these waiting time targets can offer information about trends in time, but they cannot provide conclusive evidence about whether CAMHS transformation has been successful in achieving its objective.^27^

Central direction was perceived to drive change rather than generating service-led ideas for change and transformation. The expectation would be that the less specific the policy recommendations, the greater the scope for innovative approaches to developing policy. Thus, ambiguity may allow for real innovation and experimentation. This could be the ‘best’ solution for policymakers to adopt as it leaves scope for each CAMH service to manage the local implementation of policy in terms of their own population’s needs or interests.^28^ However, what is probably even more important is to strike the right balance between the specificity of nationally prescribed and centrally imposed recommendations and local appetite for more autonomy and deliberately ambiguous policy that empowers local implementation, accommodating diversity and contextual differences.^29^ All such approaches would require close liaison with, and an understanding of, the position of the frontline practitioners delivering change locally and tailoring of recommendation to better understand what should reasonably be expected to be delivered, by whom and under what circumstances.

### Strengths and limitations

This was an exploratory study, largely descriptive in nature, and the findings reported here require further study. The sample was drawn from Scotland and the accounts presented in this paper may not sufficiently represent the views and experiences of others engaged in mental health policies and practice. Although the methods employed were as rigorous as possible, the design of the study meant that we did not speak to CYP or their carers upon whom the policies may have impacted. There is a need to broaden the sample representation to include more diverse experiences. Thus, our findings may have limited generalisability of the findings while at the same time participant narratives were very similar, suggesting that the results are likely to be consistent across a diverse range of stakeholders.

## Conclusion

Policymakers, organisations supporting the delivery of recommendations and frontline practitioners face many common challenges. There is need for an understanding that governmental, national and local drivers should to be coordinated in a whole-system approach to ensure providers deliver high quality services to meet actual needs. These findings will be pertinent more widely beyond policies focusing on improving CYP mental health.

## Supplemental Material

sj-pdf-1-hsr-10.1177_13558196211072472 – Supplemental Material for Perceived impact of formulating, implementing and enacting national mental health policies recommendations in practice: An exploratory qualitative study within child and adolescent mental health services in Scotland Click here for additional data file. Supplemental Material, sj-pdf-1-hsr-10.1177_13558196211072472 for Perceived impact of formulating, implementing and enacting national mental health policies recommendations in practice: An exploratory qualitative study within child and adolescent mental health services in Scotland by Madalina Toma, Julie Anderson, Sarah Forster, Paula Shiels, Shirley Windsor, Nicola Gray in Journal of Health Services Research & Policy
