# Supplementary material for: Perceived impact of formulating, implementing and enacting national mental health policies recommendations in practice: An exploratory qualitative study within child and adolescent mental health services in Scotland
Source: J Health Serv Res Policy. 2022 Feb 28;27(3):203–10. doi: 10.1177/13558196211072472 (PMC9277320; doi:10.1177/13558196211072472)
Supplement: sj-pdf-1-hsr-10.1177_13558196211072472 – Supplemental Material for Perceived impact of formulating, implementing and enacting national mental health policies recommendations in practice: An exploratory qualitative study within child and adolescent mental health services in Scotland [file sj-pdf-1-hsr-10.1177_13558196211072472.pdf]

Online Supplement 1

Semi-Structured Interview Topic Guide

- Can you please tell me a bit about yourself, your role and your experience to date?
- How are policies in the area of mental health and well-being currently developed and delivered?
- How does a new relevant policy gets communicated? How does your organisation gets commissioned on specific work or projects?
- What specific national policies (regulations or guidelines) are currently influencing your planned activities around improving CYP mental health? For instance, how much is your work influenced by GIRFEC, the new Mental Health Strategy or similar policies like the Scottish Government Programme for Government in Mental Health or the CYP MH Task Force Delivery Plan? What about the recent reports like the Rejected Referrals and Audit Scotland CYP mental health report? How do you navigate through all these recommendations emerging from different national regulatory bodies?
- Are you involved in any way in implementing the recommendations from the new mental health strategy which directly related CAMHS? If so, in what way? What does good practice look like and how was this new way of working developed and progressed? How was the work different from other initiatives that already existed? Was there any resistance towards this work and why did you think that was the case? Who were the main collaborators in this work and how did the collaboration come about? Is there anyone that should have been involved and was not?
- How did you know that the work had the expected results or impacts? For instance, did it have any effects on reducing the waiting times for specialist services and how useful was to have a target to achieve? How do you balance these nationally impose standards and the need for wider accountability and local ownership for CYP mental health? What trade-offs are acceptable both clinically and ethically, and what delivers the best outcomes and experience for patients?
- In what ways do recommendations from the new mental health strategy reflect the real challenges at local level? How does their content relate to local needs and priorities? How often do you think the recommendations are translated into improved practice? What specific challenges do you usually meet when trying to match service delivery and new legislation? Are these challenging specific to efforts directed towards improving mental health and well-being? Or can they be applied more broadly?
- What type of support do you receive in order to implement the specific recommendation aimed at CAMHS? What is the role of your organisation in supporting this translation process? What about the role of local authorities, social services and other third sector organisations? How do you manage local and national priorities, particularly when they are very different or even contradictory? How do you think local health boards and health and social care partnerships manage local and national priorities?

- How do you evaluate or monitor the impact of your work going forward? How often is the evaluation is fed back into local practice, national improvement agenda or wider policies? How can we better bring practice into policy?
- What are the lessons learned that can be spread to other organisations? What were the key ingredients for success and the main barriers that had to be overcome?

End: Thanks participant and ask if there are any questions or other clarification needed

Focus Group Interviews

Welcome, introductions and double-checking of consent procedures (ALL)

The constantly changing policy landscape in the area of children and young people mental health (*Facilitator 1)*

- What specific national policies (regulations or guidelines) are currently influencing the local service delivery and provision? For instance, how much is your work influenced by GIRFEC, the new Mental Health Strategy or similar policies like the Scottish Government Programme for Government in Mental Health or the CYP MH Task Force Delivery Plan? What about the recent reports like the Rejected Referrals and Audit Scotland CYP mental health report? How do local boards and health and social care partnerships navigate through all these recommendations emerging from different national regulatory bodies?
- In what ways do recommendations from the new mental health strategy (or other recent directives) towards improving CAMHS reflect the real challenges at local level? How does their content relate to local needs and priorities? How often do you think the recommendations are translated into improved practice? What specific challenges do you usually meet when trying to match service delivery and new legislation?
- What type of support do integrated local boards and health and social care partnerships receive in order to implement the specific recommendation aimed at improving CAMHS? What is the role of national organisations like the Healthcare Improvement Scotland or the Taskforce in supporting this translation process? What about the role of local authorities, social services and other third sector organisations? How do you manage local and national priorities, particularly when they are very different or even contradictory?

Comfort break

Improving CAMH Services: achievements, challenges and ways forward *(Facilitator 2)*

- There's been a lot said about the strain mental health services for young people are under. What does good practice look like and what type of initiatives are locally developed to improve the way in which services are being delivered? How was this work different from other initiatives that already existed? Was there any resistance towards this work and why did you think that was the case? Who were the main collaborators in this work and how did the collaboration come about? Is there anyone that should have been involved and was not?
- How did you know that the work had the expected results or impacts? Did it have any effects on reducing the waiting times for specialist services and how useful was to have a target to achieve? How do you balance these nationally impose standards and the need for wider accountability and local ownership for CYP mental health?

What trade-offs are acceptable both clinically and ethically, and what delivers the best outcomes and experience for patients?

- How do you evaluate or monitor the impact of your work going forward? How often is the evaluation is fed back into local practice, national improvement agenda or wider policies? How can we better bring practice into policy?
- What are the lessons learned that can be spread to other localities? What were the key ingredients for success and the main barriers that had to be overcome?

End: Thanks participants and ask if there are any questions or other clarification needed (ALL)
